# Medical and Philosophical News

**Published:** 1781-10

**Authors:** 


### SECTION III.

### Medical and Philosophical News.

THE Society of Arts and Sciences at Utrecht have propofed the following queftion for a prize of thirty ducats: " Is the planting of trees in great towns and their environs falutary, or hurtful to the health of the inhabitants; Do their exhalations purify or corrupt the air; and what fpecies of trees do the moft, or the leaft good or harm? The differtations are to be fent to M. Van-Haeften, Secretary to the Society, before the 1ft of December 1782.

---

A man, who was bit by a mad dog in the lip May 28, 1781, was attacked June 13th with the ufual fymptoms of hydrophobia. He was carried to the Hotel Dieu at Paris June 15th, where he died the fame day. After his death the whole courfe of the vertebral canal was carefully infpected, and the membranes appeared to be in an inflamed ftate. This was done at the requeft of Dr. Salin, a phyfician at Paris, who is of opinion, that in hydrophobia the medulla fpinalis is the principal feat of the difeafe.

---

The

The ſenate of Venice have lately founded an Academy of Sciences at Padua. This city, which has long been famous for its univerſity, had likewiſe two academies; that of the *Ricovrati* for the *belles lettres*, which was of long ſtanding, and that of agriculture, which was of a more recent date. Both theſe inſtitutions have been ſuppreſſed in favour of the new academy.

---

The bark of the *Daphne Laureola*, or *ſpurge laurel*, macerated in vinegar and applied to the ſkin, has of late been much employed in France and ſome other parts of Europe in caſes where the uſe of iſſues has been indicated. This acrid ſubſtance ſoon inflames and excoriates the ſkin, and if occaſionally renewed keeps up a very copious diſcharge. In the Tranſactions of the Academy of Sciences at Mentz for 1780, Dr. Rumpel recommends for a ſimilar purpoſe the bark of Mezereon, which is another plant of the ſame genus. He relates ſeveral caſes of ophthalmia, rheumatiſm, epilepſy, &c. in which it proved efficacious, when applied in the manner above deſcribed.

M. Dombay, an ingenious French botaniſt, who has been employed in Peru ever ſince the year 1777, for the purpoſe of collecting the plants and other natural productions of that country, has ſent home ſpecimens of a vegetable wool, which is ſhorter than that of the Lamas, but of the ſame colour. It is produced by a ſpecies of *Cactus Spinoſus* L. He has likewiſe analyſed the Thermal waters at Ceuchin near the Andes. When he wrote laſt to his friends in Europe, he was on the point of ſetting out on a botanic expedition to the ſource of the river of Amazons, where he expected to meet with the tree that produces the elaſtic gum. Although the country round Lima is parched for want of rain, he has ſent to the King of Spain three hundred coloured drawings of plants collected in that neighbourhood, many of which will be new to European botaniſts.

---

The Academy of St. Peterſburgh have given a premium to M. Kratzenſtein, profeſſor of chemiſtry at Copenhagen, for his very ingenious invention of a wind inſtrument that imitates diſtinctly the ſound of the vowels, and even ſome articulate ſounds.

---

At

At a general meeting of the phyficians, furgeons, and apothecaries of Liverpool on the 2d of October, it was unanimoufly refolved to recommend General Inoculation at ftated periods to the inhabitants of that town. Among other reafons for this benevolent propofal, it was obferved, that the natural fmall pox has proved fatal to more than one in five of all the children born in Liverpool, according to the obfervations of a phyfician of great eminence, lately an inhabitant of that place, and that plans of general inoculation have lately been fuccefsfully executed at Chefter and Leeds.

---

On Monday the 1ft of October the Royal College of Phyficians held their annual meeting for the choice of a Prefident and other officers, when the following gentlemen were elected:

Dr. William Pitcairn, *Prefident.*

Dr. Robert Thomlinfon, *Treafurer.*

Sir Noah Thomas, Knt.

Dr. William Cadogan,

Sir Richard Jebb, Bart.

Dr. Donald Monro, } *Cenfors.*

Dr. Henry Revell Reynolds, *Regifter.*

